# Human Stressors Are Driving Coastal Benthic Long-Lived Sessile Fan Mussel *Pinna nobilis* Population Structure More than Environmental Stressors

**DOI:** 10.1371/journal.pone.0134530

**Published:** 2015-07-28

**Authors:** Salud Deudero, Maite Vázquez-Luis, Elvira Álvarez

**Affiliations:** 1 Instituto Español de Oceanografía (IEO). Centro Oceanográfico de Baleares. Palma de Mallorca, Spain; 2 Govern de les Illes Balears. Direcció General de Medi Rural i Marí. Palma de Mallorca, Spain; University of Genova, Italy, ITALY

## Abstract

Coastal degradation and habitat disruption are severely compromising sessile marine species. The fan shell *Pinna nobilis* is an endemic, vulnerable species and the largest bivalve in the Mediterranean basin. In spite of species legal protection, fan shell populations are declining. Models analyzed the contributions of environmental (mean depth, wave height, maximum wave height, period of waves with high energy and mean direction of wave source) versus human-derived stressors (anchoring, protection status, sewage effluents, fishing activity and diving) as explanatory variables depicting *Pinna nobilis* populations at a mesoscale level. Human stressors were explaining most of the variability in density spatial distribution of fan shell, significantly disturbing benthic communities. Habitat protection affected *P*. *nobilis* structure and physical aggression by anchoring reveals a high impact on densities. Environmental variables instead played a secondary role, indicating that global change processes are not so relevant in coastal benthic communities as human-derived impacts.

## Introduction

The family Pinnidae Leach 1819 (order Pterioida) is a taxonomic family of large saltwater clams commonly known as pen- or fan-shells. These are filter-feeder marine bivalve molluscs with a long triangular shape and fragile shells with the pointed end anchored in the substrate using the byssus threads. The shells reach 15–35 cm length, exceptionally up to 120 cm [1]. This family includes two genera (*Pinna* and *Atrina*, [2]) with 61 species described worldwide [3]. Most of the species are geographically located in the Indo-Pacific area, with some inhabiting the Mediterranean Sea, Caribbean Sea, North East Atlantic, West Africa, and West America.

In the Mediterranean Sea, *Pinna nobilis* Linnaeus 1758 is the largest endemic bivalve [4] and lives at depths ranging from 0.5 to 60 m [5]. In the 20^th^ century, *P*. *nobilis* populations have greatly declined due to anthropogenic activities, including recreational and commercial fishing, ornamental harvesting, and accidental killing by anchoring, bottom nets and trawlers [6,7]. Nowadays, this species is legally protected under both Annex II of the Barcelona Convention [8] and Annex IV of the EU Habitats Directive [9]. Consequently, scientific studies to assess the current populations status in the view of future management and to set up conservation strategies are a priority. This knowledge might be extrapolated elsewhere to other species of the Pinnidae family. However, in spite of protection, *P*. *nobilis* populations are severely affected by several impacts [10–13].

The way environmental conditions and population processes determine the abundance and distribution of species is a central problem of ecology and biogeography [14]. Usually, variation in population density is a combination of several factors, such as spatial variables that affects distribution of organisms. Human-driven impacts have adverse effects on marine biota and habitats and can act determining structural and functional changes in habitats and species [15].

In this regard, human stressors might guide and modify population traits in benthic species. Previous studies already pointed out responses of *P*. *nobilis* densities to fishing mortality was much higher than natural mortality acting as important driver of the spatial distribution of this species and population viability [10]; and that the species was also served as seafood in restaurants [16]. A recent study suggested that boat anchoring in shallow waters seemed to be one of the main factors influencing *P*. *nobilis* population densities [11]. Vázquez-Luis et al.[13] experimentally demonstrated the direct impact of anchoring on *P*. *nobilis* mimic units; and other research stressed impacts of boat propellers in shallow populations [12].

The second set of environmental stressors influencing *P*. *nobilis* population includes hydrodynamic processes and storms. García-March *et al*. [17] noted that selective pressures regulate *P*. *nobilis* population parameters, producing a trade-off between hydrodynamics and shell size and orientation, for different shore types and water depth; and in shallow population living at 6 m depth hydrodynamic forces killed large individuals. Hendriks *et al*. [18] demonstrated that *P*. *oceanica* meadows provide shelter from hydrodynamic forces to *P*. *nobilis*, most appreciable for seagrass meadows located in shallow waters where the smaller animals remain within the canopy. However, in the Gulf of Oristano, wave action was not a significant factor influencing the orientation of the *P*. *nobilis* shell due to the low hydrodynamic characteristics of the area [19]. These authors demonstrated that bottom current direction and speed have a greater influence on the spatial density pattern of the bivalve compared to exposure to waves [19].

Most of the studies conducted on *Pinna nobilis* focused on density and spatial distribution along the Mediterranean Sea in a regional context, with the majority addressing coastal lagoons. Some studies have evaluated biological aspects such as the shell epibiontic community, feeding ecology, growth, recruitment, genetic structure and contaminants, among others [20]. Nevertheless, few studies had been performed including a mesoscale spatial range, and up to our knowledge, no studies had been done addressing environmental and human stressors within *Pinna nobilis* populations.

This work aims to understand the role that both environmental and human stressors play in the distribution and abundance of fan mussel *Pinna nobilis*, a sessile benthic, vulnerable and long-living species. Cumulative impacts at coastal areas are increasing and responses of key species might provide clues for management actions fostering environmental protection. This is the first study that combines and compares partial contributions of environmental and human stressors to model the effect that these variables exert on the *P*. *nobilis* population. Knowledge of key process and factors shaping endangered species and linked marine ecosystems are essential to provide appropriate management strategies for coastal species conservation.

## Methods

Parque Nacional Maritimo Terrestre del Archipielago de Cabrera provided permission for underwater surveys within the National Park. Conselleria de Medi Ambient del Govern Balear provided permission for surveying fan mussels in locations other than the National Park. Fan mussel *Pinna nobilis* is a protected species, but no extraction or manipulation was done on any of the surveyed individuals. Only scuba diving visual census for density counts and length measures estimations were carried out, not interfering with the species itself.

### Study area

**Fig 1:**
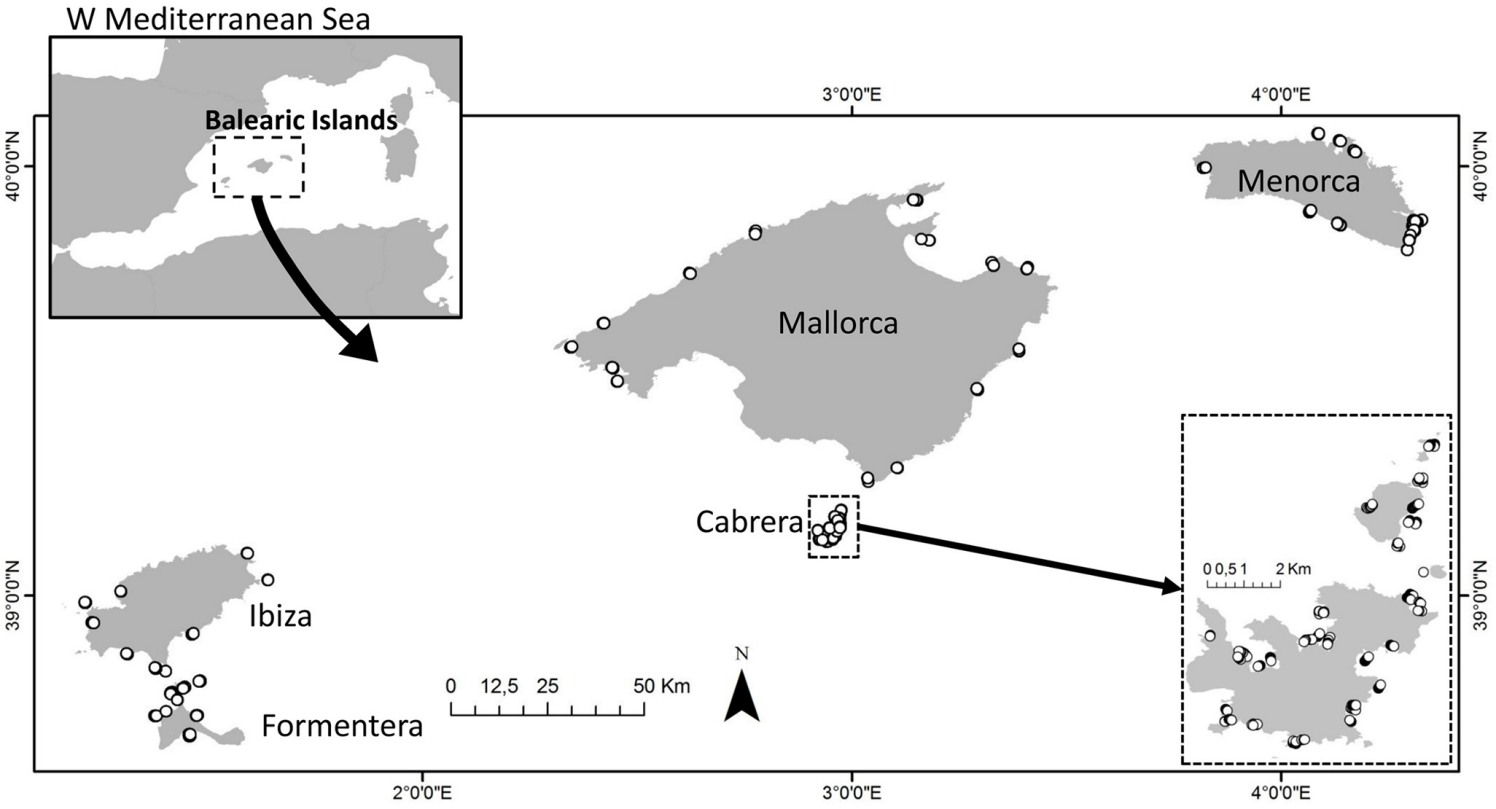
Study area. Study area in the Balearic Islands showing sampling localities (white dots) among islands.

We conducted the studies in the Balearic Islands, western Mediterranean Sea at five main islands: Mallorca, Menorca, Ibiza, Formentera and Cabrera and several islets (Fig 1). Seagrass meadows (*Posidonia oceanica* (Linnaeus) Delile, 1813) are one of the most significant elements of the benthic environment of Balearic coasts, growing on carbonate sediments of biogenic origin [21]. Extensive and dense meadows up to depths of 40 m inhabit the archipelago, but many been degraded as a result of anchoring and pollution from recreational boating, which is largely unregulated around the islands [22]. This mesoscale study integrates variability among hundreds of kilometers covering an area of around 150,000 km^2^ [23].

### Data Collection

#### *Pinna nobilis* surveys

We conducted a total of 661 visual censuses by scuba diving to survey fan mussels density in different islands of the Balearic Archipelago (Cabrera, Formentera, Ibiza, Mallorca and Menorca) at two different depths (10 and 20 m) in *Posidonia oceanica* seagrass meadows. We selected different localities at each island and depth sampling a total of 23 localities in Cabrera, 9 in Formentera, 8 in Ibiza, and 14 in Mallorca and Menorca (Fig 1). We carried out the field surveys from July 2011 to September 2013. We conducted the underwater visual censuses along strip transects of 30 m length and 2.5 m width (total area per transect of 75 m^2^ after Vazquez-Luis et al. [24]) in order to survey the fan mussel *P*. *nobilis* population density and shell size (measured as maximum shell width). We performed a total of 234 strip transects in Cabrera, 69 in Formentera, 80 in Ibiza, 140 in Mallorca and 138 in Menorca. The total surveyed surface accounting for all islands was 49,575 m^2^. We expressed *P*. *nobilis* densities as individuals per 100 m^2^.

#### Environmental and human variables

In the present study we choose 10 variables, considered as possible stressors for *P*. *nobilis* population structure (density, shell size and spatial distribution). We divided the variables in two groups (major variables): a) environmental variables which included: mean depth, Hs mean, Hs maximum, mean Tp and mean direction; and b) human variables which included: anchoring, protection status, sewage effluents, fishing activity and diving. *Hs mean* was the significant wave height, *Hs maximum* was the maximum wave height, *Mean Tp* was the period of the waves with higher energy and *Mean direction* as direction of wave source [25]. We obtained the wave data from the closest WANA node to each locality for the period 2000–2014 [25]. We divided *Anchoring* in three levels: 1-No anchoring pressure, 2-Low anchoring intensity 3-Medium anchoring intensity (with data obtained from local knowledge and historical Google images) [26]. *Protection status* comprised 5 categories from the highest to the lowest protection level: 1-National Park, 2- Marine Reserve, 3- Natural Park, 4- Sites of Community Importance (SCI) and 5- Non protected areas. Regarding sewage effluents we assigned a value of 1 when no effluents were in the area, and 2 if effluents were present in a radius of 1 km from the sampling locality [27]. We classified *Fishing activity* in two groups: with value of 1 for fishing forbidden and 2 when the activity was allowed. Finally, we categorized the *diving activity* based in expert criteria in two groups according to areas with common diving activity in a radius of 1 km: absence-1 and presence-2.

### Data analyses

#### Spatial variation of fan mussel *P*. *nobilis* population

We performed a Permutational multivariate analysis of variance (PERMANOVA) to test differences on: living *P*. *nobilis* densities (ind/100m^2^), maximum shell width of living individuals (cm), dead *P*. *nobilis* densities, maximum shell width of dead individuals; and percentage of living and dead *P*. *nobilis* individuals (excluding transects with zero individuals for percentage data) across islands, depths and localities. The experimental design comprised 3 factors: “Island” fixed with 5 levels of variation: Mallorca, Menorca, Cabrera, Ibiza and Formentera; “Depth” fixed with two levels of variation: 10 and 20 meters; and “Locality” random and nested with both main factors. We transformed data on *Pinna* densities to fourth root, while non-transformed data were analyzed for shell size and percentage. We applied a Bray-Curtis similarity to fan mussel densities and shell size, while Euclidean distance was calculated for percentage data with 999 permutations. We examined significant terms in the full model applying a posterior pair wise comparisons [28]. We calculated all statistical analyses using software PRIMER v6 [29] with the add-on package PERMANOVA+ [30].

#### Relationship between fan mussel *P*. *nobilis* population and environmental and human variables

We applied a distance-based linear model routine (DistLM) in order to test relationships among tested variables: living *P*. *nobilis* densities, maximum shell width of living individuals, and percentage of dead *P*. *nobilis* individuals (considered as a proxy of a rate of mortality) with environmental and human stressors (explanatory variables) [31]. The analysis was based on log (x+1) data transformed for densities, and untransformed data for size and mortality; and in all cases similarity matrices were constructed using Euclidean Distance. Additionally, for living *P*. *nobilis* density we conducted a DistLM analysis based on major variables (environmental versus human) based on log (x+1) data transformed and Euclidean Distances. In all cases, we applied the method specified selection and adjusted R^2^ criterion with 999 permutations. We calculated draftsman plots with the corresponding Pearson correlation coefficients to explore the relationships between the environmental and human variables [29] and to detect strongly correlated variables. We excluded no variable due to low correlations between them. We plotted the output of the dbRDA axis and the tested variable through a PCA, superimposing the original regressor variables as vectors (multiple correlation) in order to visualize the relationships between tested variables and explanatory variables (we analyzed a matrix of a single tested variable: Anderson, *pers*. *comm*.). We performed all statistical analyses with PRIMER v6 [29] with the add-on package PERMANOVA+ [30].

## Results

### Fan mussel *P*. *nobilis* populations

**Fig 2:**
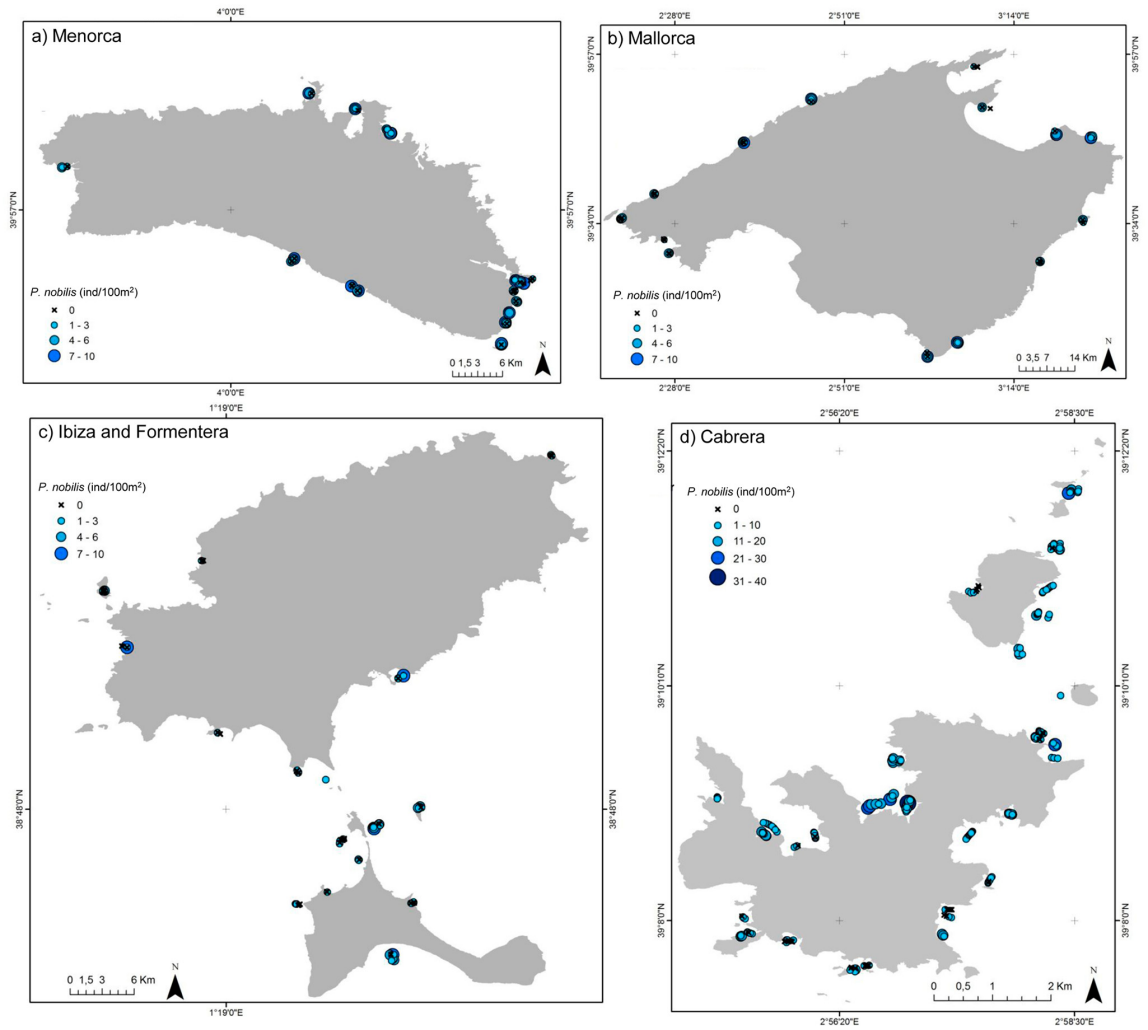
Density of *P*. *nobilis* populations. Densities of living *P*. *nobilis* (ind/100m^2^) found across islands and localities. (Note that density scale differs for Cabrera).

**Fig 3:**
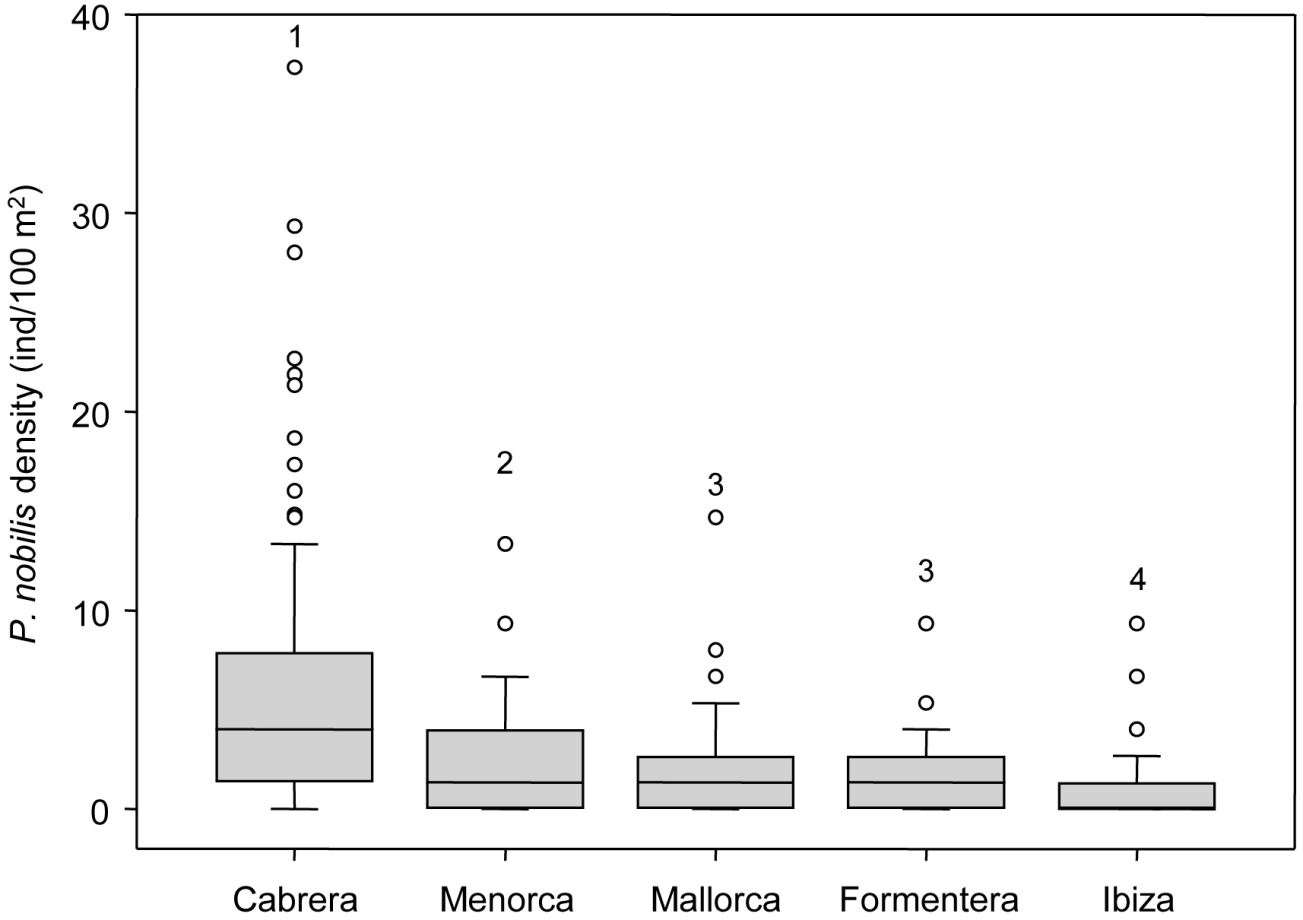
Mean values (±standard error) of living *P*. *nobilis* density (individuals/100m^2^) among islands. Median values are included, the rectangles contain values between the first and the third quartiles, the bars represent the variability outside the upper and lower quartiles, outliers are shown as circles. SNK groups are shown by numbers.

We recorded a total of 2081 specimens of *P*. *nobilis* by visual census along 49,575 m^2^ surveyed. Regarding spatial allocation among islands, we censed 1328 individuals in Cabrera, 113 in Formentera, 70 in Ibiza, 247 in Mallorca and 323 in Menorca. Overall dead individuals represent 17.78% of the counted fan shell, with relative values among the islands of 18.5%, 19.5%, 30%, 15.8% and 13%, respectively. Total observed average densities among islands were 3.21±0.13 ind/100 m^2^, varying among islands and among localities (Fig 2, for further details see S1 Table). Regarding living individuals, we found the highest density values in Cabrera 5.74 value ind/100 m^2^ and the lowest in Ibiza 0.83 mean ind/100 m^2^ (Fig 3, Table 1, Is: *p* <0.01).

**Table 1:** Results of the PERMANOVA analysis for data on *P*. *nobilis* density, shell size and % of presence.

	**Living *P*. *nobilis* density**			**Living *P*. *nobilis* size**			**Dead *P*. *nobilis* density**		
**Source**	df	MS	*P* (perm)	df	MS	*P* (perm)	df	MS	*P* (perm)
**Is**	4	11.98	0.0001**	4	2287.6	0.003**	4	2.96	0.001**
**De**	1	1.34	0.2523	1	15396	0.001**	1	0.81	0.207
**IsxDe**	4	0.86	0.5016	4	645.34	0.253	4	0.53	0.36
**Lo(IsxDe)**	111	1.15	0.0001**	101	1042.8	0.001**	111	0.52	0.001**
**Residual**	540	0.25		1599	175.79		540	0.25	
**Total**	660			1709			660		
**SNK**	Is: C ≠ Me ≠ Ma = F ≠ I			Is: C = Me ≠ Ma, F, I; De: 20 ≠ 10			Is: C ≠ Me, Ma, F, I		
	**Dead *P*. *nobilis* size**			**% Living *P*. *nobilis***			**% Dead *P*. *nobilis***		
**Source**	df	MS	*P* (perm)	df	MS	*P* (perm)	df	MS	*P* (perm)
**Is**	4	3100.5	0.001**	4	716.98	0.186	4	10236	0.001**
**De**	1	13134	0.001**	1	12.26	0.879	1	316.03	0.54
**IsxDe**	4	2026.4	0.001**	4	585.11	0.326	4	1037.3	0.384
**Lo(IsxDe)**	82	480.57	0.001**	101	553.3	0.001**	82	1239.6	0.001**
**Residual**	278	146.33		336	287.14		126	418.88	
**Total**	369			446			217		
**SNK**	IsxDe: C, F, I: 20≠10;Ma, Me: 20 = 10						Is: C ≠ Ma, F, I; I ≠ Ma, Me, F		

Data on living *P*. *nobilis* density (individuals/100m^2^), of living *P*. *nobilis* shell size (maximum width), dead *P*. *nobilis* density, dead *P*. *nobilis* shell size, and % of living and dead *P*. *nobilis* per transect (without transects with zero individuals).

Is = Islands; De = Depth; Lo = Locality; df = degrees of freedom; MS = mean square; *P* = level of significance; C = Cabrera; Me = Menorca; Ma = Mallorca; F = Formentera and I = Ibiza.

** = significant (*P*< 0.01).

**Fig 4:**
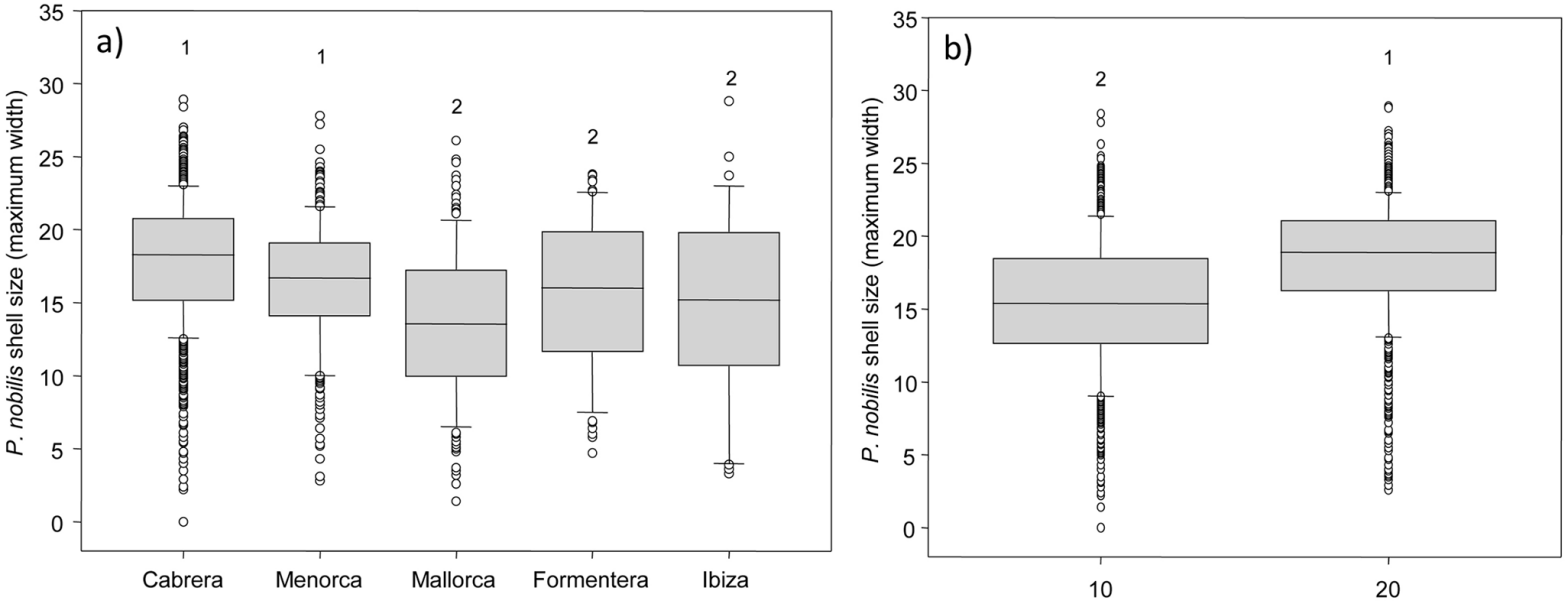
Mean values of *P*. *nobilis* shell size across: a) Islands and b) Depth. Mean values (±standard error) of living *P*. *nobilis* shell size (maximum width). Median values are included, the rectangles contain values between the first and the third quartiles, the bars represent the variability outside the upper and lower quartiles, outliers are shown as circles. SNK groups are shown by numbers.

In terms of shell size structure we found significant differences for both main factors island and depth. Regarding factor island, *P*. *nobilis* shells were bigger in Cabrera and Menorca and smaller in Mallorca, Formentera and Ibiza (Fig 4a, Table 1, Is: *p* <0.01). With respect to depth, individuals were bigger at 20 meters depth (Fig 4b, Table 1, De: *p* <0.01; for further details on size structure see S1 Fig).

Regarding dead individuals, density was higher in the protected island of Cabrera (Table 1, Is: *p* <0.01) and shell sizes of dead individuals were different across islands and depths. At Cabrera, Formentera and Ibiza shell size were bigger at 20 meters depth (Table 1, IsxDe: *p* <0.01). Regarding ratio of living and dead *P*. *nobilis* density, percentage of living individuals varied among localities; however, percentage of dead individuals (such a rate of mortality) was significant different among islands being higher in Ibiza (Table 1, Is: *p* <0.01).

### Relationship between fan mussel *P*. *nobilis* population and environmental and human variables

**Fig 5:**
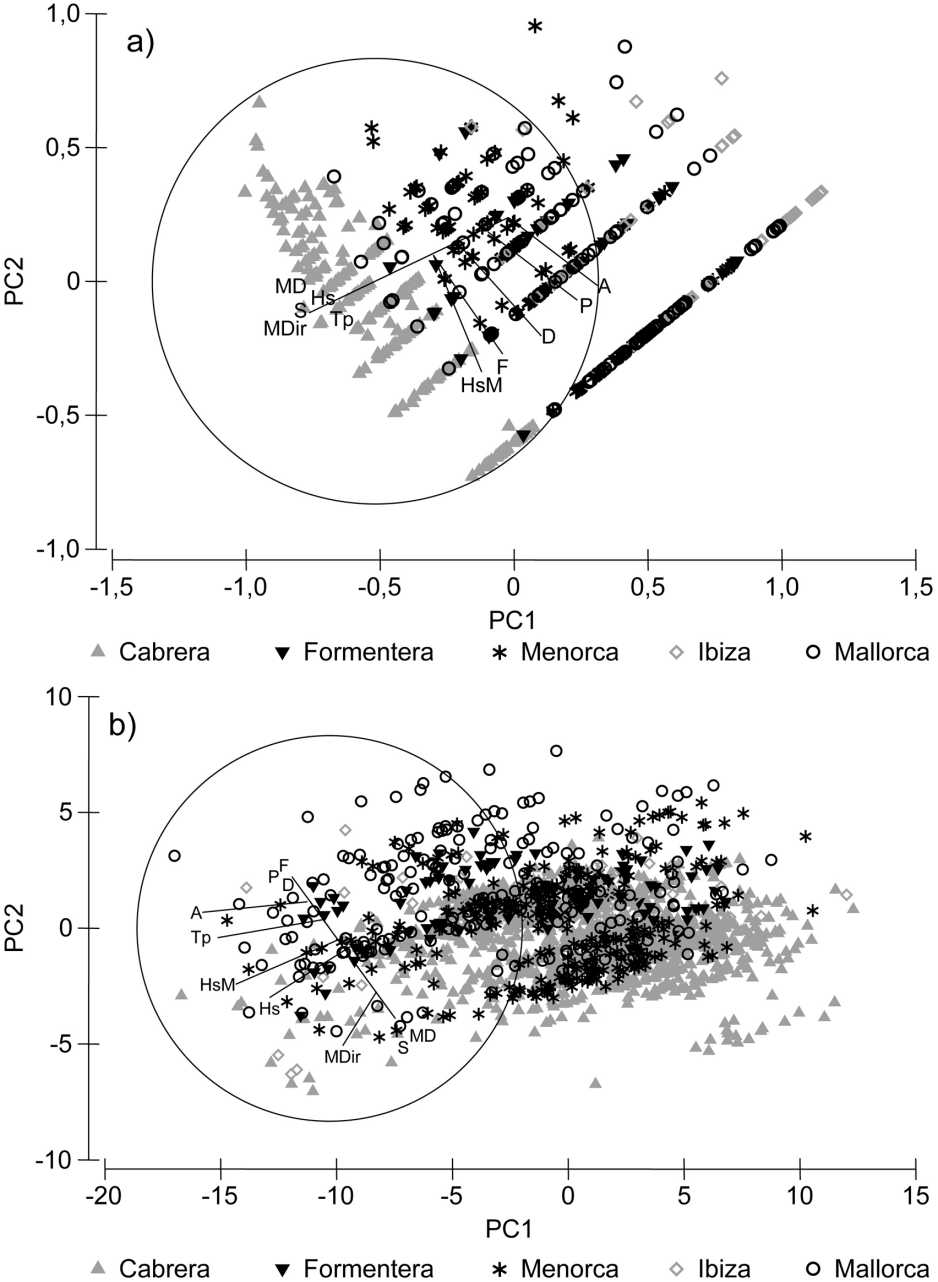
Relationship between the ordination of (A) living *P*. *nobilis* densities, and (B) living *P*. *nobilis* size, and environmental and human variables. Environmental variables: MD: mean depth, Hs: Hs mean, HsM: Hs maximum, Tp: mean Tp, MDir: mean direction. Human variables: A: anchoring, P: protection status, S: sewage effluents, F: fishing activity and D: diving.

**Fig 6:**
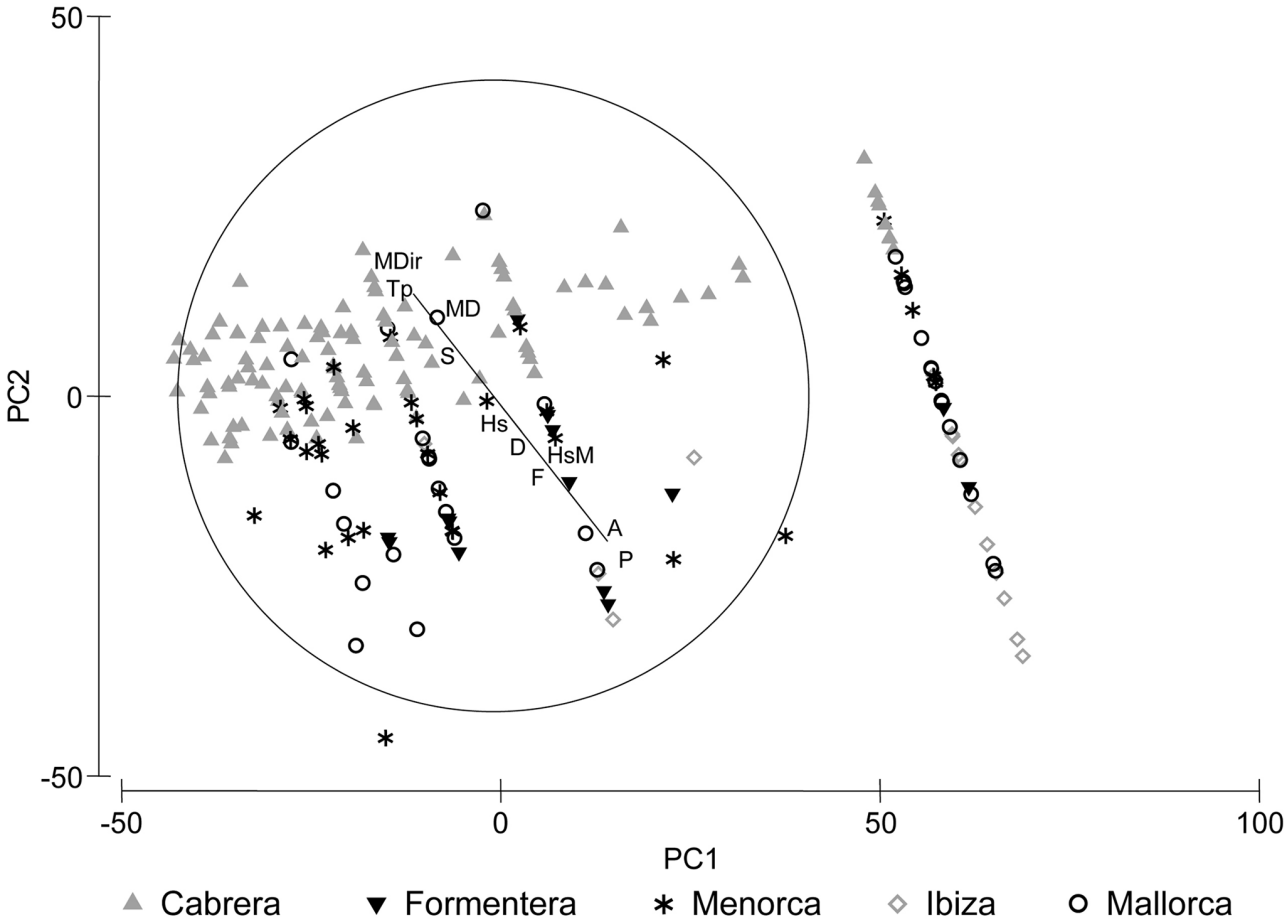
Relationship between the ordination of *P*. *nobilis* mortality (% dead *P*. *nobilis* individuals) and environmental and human variables. Environmental variables: MD: mean depth, Hs: Hs mean, HsM: Hs maximum, Tp: mean Tp, MDir: mean direction. Human variables: A: anchoring, P: protection status, S: sewage effluents, F: fishing activity and D: diving.

The DistLM analysis calculated on living *P*. *nobilis* density showed significant relationship with major variables. The model exhibited the highest percentages of variability explained by the group of human stressors (21.6%), while environmental variables explained only 6.6% of variability in the model (Table 2, Fig 5a). Moreover, DistLM analysis for living *P*. *nobilis* density contrasted with the ten explanatory variables showed significant relationships. The highest percentages of variability were explained by anchoring (19.92%) followed by protection status (11.71%); being also significant in the model the mean wave direction, fishing activity and diving. The model explained altogether the 22.93% of variation. Regarding living *P*. *nobilis* shell sizes the overall explained variation was 24.16%, being the most contributing variables mean depth, mean direction and anchoring (Table 2, Fig 5b). The model explained the 26.18% of variability in the mortality ratio (% dead individuals). Considering the variables individually, anchoring (17.29%) and protection status (13.71%) explained the highest percentage of variability (Table 3, Fig 6). In all cases, the less contributing variables were maximum wave height Hs, sewage effluents and mean period of the waves with higher energy Tp.

**Table 2:** Results of DistLM for: a) living *P*. *nobilis* densities, major variables; b) living *P*. *nobilis* densities; and c) living *P*. *nobilis* sizes.

**a) Living *P*. *nobilis* densities**			
**Major Variable**	**Pseudo-*F***	***p***	**% var**.
Environmental	9.2603	0.001	6.602
Human	36.039	0.001	21.575
**b) Living *P*. *nobilis* densities**			
**Variable**	**Pseudo-*F***	***p***	**% var**.
Mean depth	0.0079	0.9658	<0.01
Hs mean	4.3023	0.0204	0.81
Hs maximum	0.8431	0.3182	0.16
Mean Tp	0.9655	0.2789	0.18
Mean direction	13.579	0.0001	2.55
Anchoring	106.16	0.0001	19.93
Protection status	62.412	0.0001	11.72
Sewage effluents	0.1216	0.7044	<0.01
Fishing activity	23.343	0.0001	4.38
Diving	32.255	0.0001	6.05
**c) Living *P*. *nobilis* size**			
**Variable**	**Pseudo-*F***	***p***	**% var**.
Mean depth	130.6	0.01	7.1
Hs mean	3.12	0.072	0.18
Hs maximum	1.063	0.304	<0.01
Mean Tp	28.117	0.001	1.62
Mean direction	71.3	0.001	4.007
Anchoring	69.656	0.001	3.918
Protection status	58.214	0.001	3.296
Sewage effluents	15.904	0.001	0.922
Fishing activity	68.897	0.001	3.877
Diving	22.985	0.001	1.33

DistLM (distance-based linear model routine) marginal test for relationships between environmental and human variables. Environmental variables: mean depth, Hs mean, Hs maximum, mean Tp and mean direction. Human variables: anchoring, protection status, sewage effluents, fishing activity and diving.

**Table 3:** Results of DistLM for mortality (percentage of dead *P*. *nobilis*).

Mortality			
Variable	Pseudo-*F*	*p*	% var.
Mean depth	1.501	0.216	0.69
Hs mean	1.5502	0.206	0.71
Hs maximum	2.5205	0.119	1.153
Mean Tp	0.0024	0.889	0.01
Mean direction	10.209	0.002	4.513
Anchoring	45.156	0.001	17.29
Protection status	34.314	0.001	13.71
Sewage effluents	0.953	0.337	0.44
Fishing activity	8.434	0.004	3.76
Diving	5.653	0.016	2.55

DistLM (distance-based linear model routine) marginal test for relationships between environmental and human variables. Environmental variables: mean depth, Hs mean, Hs maximum, mean Tp and mean direction. Human variables: anchoring, protection status, sewage effluents, fishing activity and diving).

## Discussion

In the Mediterranean Sea cumulative impacts of human-derived activities are steering biodiversity loss. Endemic and vulnerable species are the target of those impacts with irreversible consequences on biological fitness and species survival. Coastal degradation, especially in high touristic areas is of high concern for vulnerable species. Some species such as the largest bivalve within the Mediterranean Sea are under high pressure [20]. Understanding drivers of species losses and ultimately extinctions are crucial to assure their conservation.

This large-scale study performed with a high spatial resolution demonstrates that the spatial distribution of *Pinna nobilis* is affected by human stressors more than environmental variables. Anchoring is the main factor affecting density of the fan mussel in the studied sites. The anchoring effect on *P*. *nobilis* densities have been already stated in previous works [11, 13]. Legal protection of habitats is crucial in maintaining population structure of large, long-lived and sessile benthic organisms such as the fan mussels. This study shows that protection is widely affecting densities of *P*. *nobilis* at the studied geographical extent. Contrasted densities are two-fold in the MPA (Cabrera) where no-take reserves have been effectively set for more than 20 years. Those results indicate that MPAs guarantee conservation demonstrating that a combination of protection size and age of the MPAs [32, 33] seems to set the optimal conditions for growth and development of the species.

Mean densities in this study are within the range of previous studies [34–36, 11], indicating that protection through MPA establishment represents a highly effective measure for conservation of this species. The same states for the regression of seagrass beds *Posidonia oceanica*, preferential habitat for *Pinna nobilis*, which degradation has been vastly documented to be under regression [37,38].

Density-dependent processes are probably limiting dispersion of propagules of the fan mussel. There might be a threshold of minimum conspecific densities of *P*. *nobilis* in the seabed to ensure gamete release and fecundity probabilities within the water masses. In addition, reproductive aspects of the fan mussel’s densities might induce larval recruitment nearby existing adults by chemical clues and ensuring juvenile replenishment [39]. Those aspects reinforce the need to keep and maintain certain density levels to guarantee population survival while ensuring genetic variability.

Eutrophication does not modify spatial distribution of the species as observed by the low variability explained by the sewage factor. Instead, sewage effluents increase nutrient loads in the oligotrophic waters of the Balearics boosting growth at juvenile stages, as previously demonstrated by stable isotopes signatures [40].

Conversely, fisheries alter fan mussel size and densities. Artisanal fisheries, mainly trammel nets entangle fan mussel’s shells and dislodge the individuals from the benthic substrate as already reported in Greece [10]. Specific management measures to avoid those conflicts could be to exclude fisheries at the extent of the *P*. *nobilis* distribution only allowing fisheries activities at the lower limit of its distribution as it is the case for *Posidonia oceanica* seagrass meadows.

Diving affects density of fan mussels in this study. Many authors have demonstrated diving impacts on sessile organisms corals, bryozoans, gorgonians [41,42]. The studied *P*. *nobilis* populations inhabit seagrass beds, and very little work has been carried out on diving impacts on seagrass and soft-bottom habitats. In our case, diving along with poaching disturbance might be altering fan mussel’s spatial distribution and densities. Foster of environmental education programs addressing conservation issues and getting to know protected species to tourist and society might help to reduce impacts of recreational activities (mainly boating and diving) on sessile organism in coastal shores.

The way to assess population structure should be carefully considered. In the studied fan mussels populations, density and size respond to different drivers. Interesting results highlight that *P*. *nobilis* densities are tightly linked to human-driven activities, while size of the individuals are responding to environmental variables. Anchoring and protection mainly drive densities, while shell size are more influenced by depth and wave direction. Additionally, significantly larger individuals are found at deeper depths, indicating cross-effects with hydrodynamics. Those responses follow previous data on shell orientation and distribution and depth effects [17].

Climate change and increase in storms and wave intensity might disturb fan mussels by dislodging the individuals from the seabottom and impinging large individuals to deeper depths. Instead, bays and sheltered areas might be protected from intense wave action [19] along with minimum thresholds in wave intensity and direction for *Pinna* shell hydrodynamics [18].

Our results prove that activities related with recreational activities (anchoring, protection, diving, fishing) have large effects on species distribution. Future scenarios at coastal areas depict a high increase in coastal activities disturbing the habitats and ecosystems. This study reveals that ensuring fan mussels populations in coastal habitats needs a wise spatial management, linking MPAs with the establishment comprehensive measures like no anchoring areas or regulated permanent mooring systems [43, 13] to avoid free anchoring. The results suggest that fan mussel *P*. *nobilis* population structure (size, densities and spatial distribution patterns) can be properly considered precise indicators of human impacts in coastal habitats by reflecting human derived activities and cumulative impacts along with environmental responses.

## Supporting Information

S1 Fig Size class of *P*.*nobilis* populations in Balearic Islands. Percentage of individuals of each size classes among islands (maximum shell width).
(TIF)

S1 Table Data *P*.*nobilis* populations in Balearic Islands. Mean density, minimum and maximum density of living and dead *P*. *nobilis* (ind/100m^2^); mean percentage, minimum and maximum percentage of living and dead *P*. *nobilis* (ind/100m^2^) among islands, localities and depth (table also includes mean depth in strata). N = number of strip transects.
(DOCX)
