# Supplementary figures and images for: Human Stressors Are Driving Coastal Benthic Long-Lived Sessile Fan Mussel Pinna nobilis Population Structure More than Environmental Stressors
Source: PLoS One. 2015 Jul 28;10(7):e0134530. doi: 10.1371/journal.pone.0134530 (PMC4517765; doi:10.1371/journal.pone.0134530)

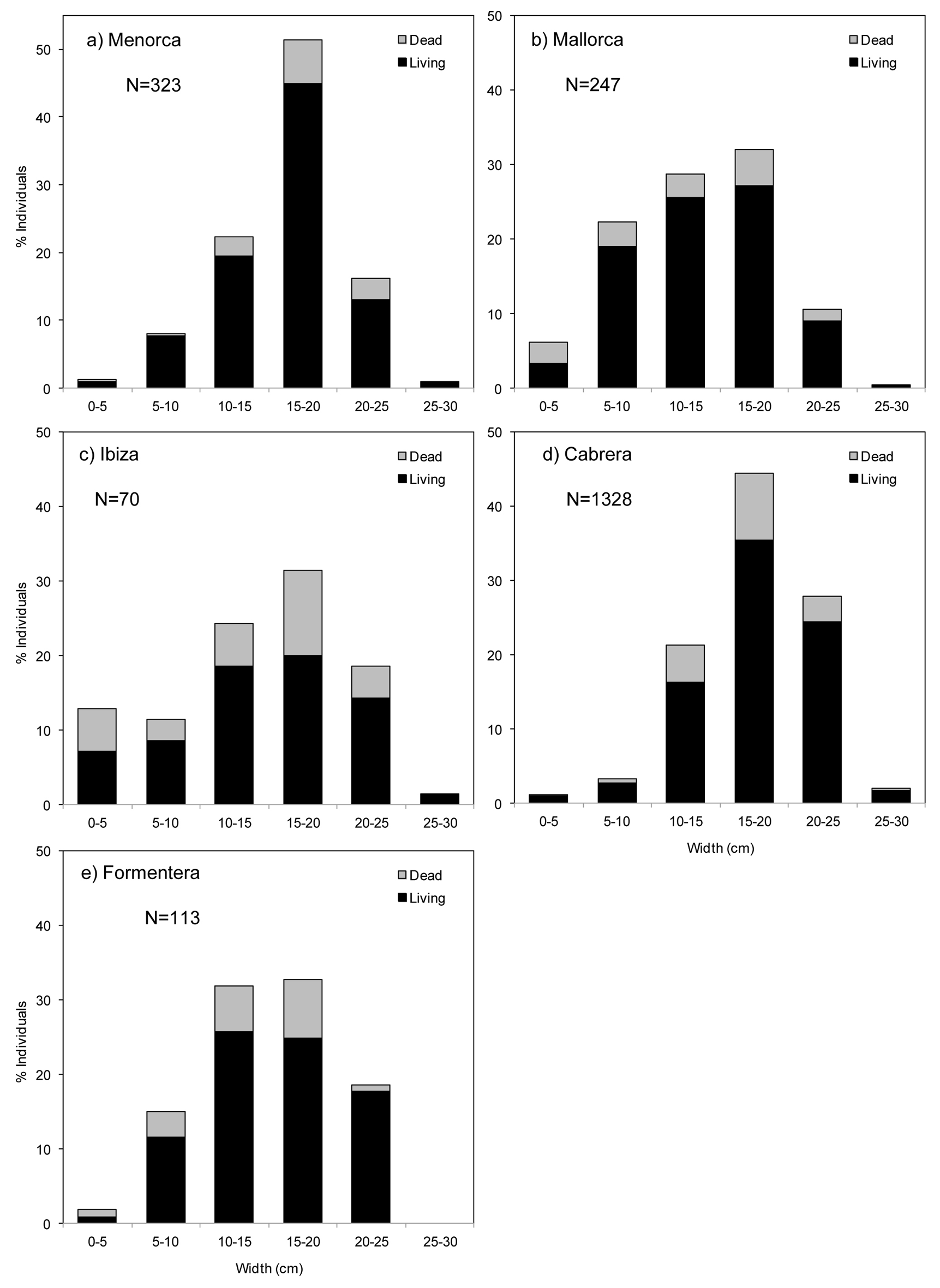

Supplement: S1 Fig — Percentage of individuals of each size classes among islands (maximum shell width). (TIF) [file pone.0134530.s001.tif]
