# Supplementary material for: Human Stressors Are Driving Coastal Benthic Long-Lived Sessile Fan Mussel Pinna nobilis Population Structure More than Environmental Stressors
Source: PLoS One. 2015 Jul 28;10(7):e0134530. doi: 10.1371/journal.pone.0134530 (PMC4517765; doi:10.1371/journal.pone.0134530)
Supplement: S1 Table — Mean density, minimum and maximum density of living and dead P. nobilis (ind/100m2); mean percentage, minimum and maximum percentage of living and dead P. nobilis (ind/100m2) among islands, localities and depth (table also includes mean depth in strata). N = number of strip transects. (DOCX) [file pone.0134530.s002.docx]

| **Island** | **Locality** | **Depth** | **N** | **Mean depth** | **Living *P. nobilis*** | | **Dead *P. nobilis*** | | **Mean % of**  **living *P. nobilis*** | **% living *P. nobilis***  **(Min-Max)** | **Mean % of**  **dead *P. nobilis*** | **% dead *P. nobilis***  **(Min-Max)** |
| --- | --- | --- | --- | --- | --- | --- | --- | --- | --- | --- | --- | --- |
|  |  |  |  |  | **(mean density)** | **(Min-Max)** | **(mean density)** | **(Min-Max)** |  |  |  |  |
| **Cabrera** | Burri | 10 | 10 | 9,1 | 4,67 ± 0,7 | 1,33 - 8 | 0,53 ± 0,22 | 0 - 1,33 | 90,84 ± 3,82 | 75,01 - 100 | 9,16 ± 3,82 | 0 - 24,99 |
|  |  | 20 | 10 | 20,9 | 3,6 ± 0,77 | 0 - 6,67 | 1,2 ± 0,54 | 0 - 5,33 | 78,93 ± 6,63 | 50 - 100 | 21,07 ± 6,63 | 0 - 50 |
|  | Cala Galiota | 20 | 5 | 15,4 | 5,6 ± 1,06 | 4 - 9,33 | 0,27 ± 0,27 | 0 - 1,33 | 95,01 ± 5 | 75,01 - 100 | 4,99 ± 4,99 | 0 - 24,99 |
|  | Cap Ventós-Dimoni | 10 | 3 | 10,6 | 2,42 ± 1,15 | 0,15 - 3,78 | 0,51 ± 0,51 | 0 - 1,52 | 90,48 ± 9,52 | 71,43 - 100 | 9,52 ± 9,52 | 0 - 28,57 |
|  |  | 20 | 3 | 20,1 | 11,11 ± 5,65 | 2,78 - 21,87 | 0 | - | 100 ± 0 | 100 - 100 | 0 ± 0 | 0 - 0 |
|  | Casa Rei | 10 | 8 | 5,4 | 2,67 ± 0,67 | 0 - 5,33 | 0,5 ± 0,24 | 0 - 1,33 | 80,83 ± 12,37 | 0 - 100 | 19,17 ± 12,37 | 0 - 100 |
|  | Conejera | 10 | 5 | 9,6 | 5,33 ± 1,63 | 1,33 - 10,67 | 2,13 ± 1,08 | 0 - 5,33 | 74,28 ± 15,2 | 19,99 - 100 | 25,72 ± 15,2 | 0 - 80,01 |
|  |  | 20 | 4 | 17,5 | 6,33 ± 1,14 | 4 - 9,33 | 3,67 ± 0,64 | 2,67 - 5,33 | 62,93 ± 4,6 | 50 - 71,43 | 37,07 ± 4,6 | 28,57 - 50 |
|  | Conejera SE | 10 | 2 | 10,7 | 9,07 ± 5,74 | 3,33 - 14,81 | 0 | - | 100 ± 0 | 100 - 100 | 0 ± 0 | 0 - 0 |
|  |  | 20 | 3 | 19,7 | 6,92 ± 3,04 | 1,26 - 11,67 | 1,69 ± 0,74 | 0,21 - 2,5 | 81,66 ± 2,56 | 76,92 - 85,71 | 18,34 ± 2,56 | 14,29 - 23,08 |
|  | Conejera-poniente | 10 | 2 | 10,7 | 0 | - | 0 | - | - | 0 - 0 | - | 0 - 0 |
|  |  | 20 | 5 | 19,4 | 1,6 ± 0,65 | 0 - 2,67 | 0,27 ± 0,27 | 0 - 1,33 | 88,89 ± 8,6 | 66,68 - 100 | 11,11 ± 8,6 | 0 - 33,33 |
|  | Cova Calamar | 10 | 9 | 9,2 | 0,44 ± 0,31 | 0 - 2,67 | 0,3 ± 0,23 | 0 - 2,67 | 75 ± 11,79 | 50 - 100 | 25 ± 11,79 | 0 - 50 |
|  | Cova Rotja | 10 | 9 | 7,6 | 11,56 ± 1,59 | 6,67 - 17,33 | 1,48 ± 0,68 | 0 - 5,33 | 90,43 ± 4,08 | 71,43 - 100 | 9,57 ± 4,08 | 0 - 28,57 |
|  |  | 20 | 8 | 18,3 | 3,33 ± 0,76 | 1,33 - 6,67 | 0,67 ± 0,36 | 0 - 2,67 | 87,5 ± 6,86 | 50 - 100 | 12,5 ± 6,86 | 0 - 50 |
|  | Dimoni | 10 | 10 | 8,7 | 5,2 ± 1,87 | 0 - 16 | 0,8 ± 0,36 | 0 - 2,67 | 88,5 ± 5,48 | 50 - 100 | 11,5 ± 5,48 | 0 - 50 |
|  |  | 20 | 8 | 18,3 | 3,5 ± 0,97 | 0 - 8 | 0,67 ± 0,25 | 0 - 1,33 | 74,53 ± 12,58 | 0 - 100 | 25,47 ± 12,58 | 0 - 100 |
|  | Estells | 10 | 10 | 6,2 | 0,26 ± 0,18 | 0 - 1,33 | 0,8 ± 0,36 | 0 - 2,67 | 26,67 ± 13,74 | 0 - 100 | 73,34 ± 13,74 | 0 - 100 |
|  |  | 20 | 8 | 18,4 | 4,33 ± 1,89 | 0 - 16 | 1,33 ± 0,76 | 0 - 5,33 | 63,89 ± 15,1 | 0 - 100 | 36,11 ± 15,09 | 0 - 100 |
|  | Estels-Ensiola | 20 | 2 | 19,2 | 5,23 ± 2,27 | 2,96 - 7,5 | 0,33 ± 0,09 | 0,25 - 0,42 | 93,52 ± 1,21 | 92,31 - 94,73 | 6,48 ± 1,21 | 5,27 - 7,69 |
|  | Freus | 10 | 10 | 9,0 | 3,2 ± 0,57 | 1,33 - 6,67 | 1,6 ± 0,39 | 0 - 4 | 69,69 ± 5,69 | 50 - 100 | 30,31 ± 5,69 | 0 - 50 |
|  |  | 20 | 8 | 16,1 | 7,83 ± 0,89 | 4 - 12 | 6,67 ± 1,28 | 2,67 - 12 | 55,62 ± 5,49 | 37,49 - 81,82 | 44,38 ± 5,49 | 18,18 - 62,5 |
|  | Freus2 | 10 | 10 | 11,2 | 3,73 ± 1,12 | 0 - 12 | 5,87 ± 1,21 | 0 - 10,67 | 39,95 ± 9,69 | 0 - 100 | 60,05 ± 9,69 | 0 - 100 |
|  | Imperial | 10 | 5 | 11,7 | 0,27 ± 0,27 | 0 - 1,33 | 0,27 ± 0,27 | 0 - 1,33 | 50 ± 31,62 | 0 - 100 | 50 ± 31,62 | 0 - 100 |
|  |  | 20 | 8 | 16,6 | 6,83 ± 2,2 | 0 - 16 | 0,33 ± 0,33 | 0 - 2,67 | 97,62 ± 2,06 | 85,71 - 100 | 2,38 ± 2,06 | 0 - 14,29 |
|  | La Olla | 10 | 10 | 8,5 | 6,13 ± 1,09 | 2,67 - 12 | 0,53 ± 0,22 | 0 - 1,33 | 91,5 ± 3,73 | 75,01 - 100 | 8,49 ± 3,73 | 0 - 25 |
|  | Moruna March | 10 | 5 | 10,0 | 6,13 ± 1,87 | 2,67 - 13,33 | 0,27 ± 0,27 | 0 - 1,33 | 98,18 ± 1,82 | 90,91 - 100 | 1,82 ± 1,82 | 0 - 9,09 |
|  |  | 20 | 5 | 18,7 | 11,47 ± 1,16 | 8 - 14,67 | 2,4 ± 0,5 | 1,33 - 4 | 83,06 ± 2,74 | 72,73 - 88,89 | 16,94 ± 2,74 | 11,11 - 27,27 |
|  | Pobra | 10 | 5 | 10,0 | 10,93 ± 3,61 | 4 - 22,67 | 0,8 ± 0,33 | 0 - 1,33 | 87,67 ± 5,21 | 75,01 - 100 | 12,33 ± 5,21 | 0 - 25 |
|  |  | 20 | 4 | 18,9 | 7,33 ± 0,86 | 5,33 - 9,33 | 1 ± 0,33 | 0 - 1,33 | 87,26 ± 4,4 | 80,01 - 100 | 12,74 ± 4,4 | 0 - 20 |
|  | Ratas 1 | 20 | 4 | 20,4 | 4,33 ± 2,13 | 0 - 8 | 0,33 ± 0,33 | 0 - 1,33 | 95,24 ± 4,12 | 85,72 - 100 | 4,76 ± 4,12 | 0 - 14,28 |
|  | Ratas 2 | 10 | 6 | 7,9 | 2,44 ± 0,72 | 0 - 5,33 | 0,44 ± 0,28 | 0 - 1,33 | 89,34 ± 6,26 | 66,68 - 100 | 10,66 ± 6,26 | 0 - 33,325 |
|  |  | 20 | 4 | 18,2 | 13 ± 2,27 | 9,33 - 18,67 | 1 ± 0,33 | 0 - 1,33 | 93,13 ± 2,59 | 87,5 - 100 | 6,87 ± 2,6 | 0 - 12,5 |
|  | Rodona (Oeste) | 20 | 1 | 19,0 | 3,49 | 3,49 - 3,49 | 0,159 | 0,16 - 0,16 | - | 95,65 - 95,65 | - | 4,35 - 4,35 |
|  | Santa Maria | 10 | 10 | 8,0 | 13,07 ± 2,98 | 2,67 - 37,33 | 2,01 ± 0,41 | 0 - 4 | 82,99 ± 3,91 | 66,68 - 100 | 17,01 ± 3,91 | 0 - 33,33 |
|  |  | 20 | 10 | 18,6 | 16,93 ± 2,34 | 8 - 29,33 | 2,13 ± 0,85 | 0 - 9,33 | 89,55 ± 2,69 | 75 - 100 | 10,45 ± 2,69 | 0 - 25,01 |
|  | S'Espalmador | 10 | 5 | 6,6 | 2,13 ± 0,68 | 0 - 4 | 0,53 ± 0,33 | 0 - 1,33 | 81,25 ± 10,7 | 50 - 100 | 18,75 ± 10,7 | 0 - 50 |
| **Formentera** | Caló de s'Oli | 10 | 3 | 7,0 | 0,44 ± 0,44 | 0 - 1,33 | 0 | - | - | 100 - 100 | - | 0 - 0 |
|  | Cargoler | 10 | 1 |  | 2,67 | 2,67 - 2,67 | 0 | - | - | 100 - 100 | - | 0 - 0 |
|  | Espalmador Llevant | 10 | 5 | 10,5 | 4,53 ± 1,24 | 2,67 - 9,33 | 0 | - | 100 ± 0 | 100 - 100 | 0 ± 0 | 0 - 0 |
|  |  | 20 | 5 | 19,0 | 2,4 ± 0,65 | 0 - 4 | 0,8 ± 0,33 | 0 - 1,33 | 77,08 ± 7,05 | 66,67 - 100 | 22,92 ± 7,05 | 0 - 33,33 |
|  | Espardell | 10 | 5 | 9,7 | 2,4 ± 0,5 | 1,33 - 4 | 0,27 ± 0,27 | 0 - 1,33 | 90 ± 10 | 50 - 100 | 10 ± 10 | 0 - 50 |
|  |  | 20 | 5 | 19,5 | 2,4 ± 0,85 | 0 - 5,33 | 0,27 ± 0,27 | 0 - 1,33 | 87,5 ± 11,18 | 50 - 100 | 12,5 ± 11,18 | 0 - 50 |
|  | Gastabí | 10 | 5 | 11,0 | 0,27 ± 0,27 | 0 - 1,33 | 0 | - | - | 100 - 100 | - | 0 - 0 |
|  |  | 20 | 5 | 20,7 | 1,07 ± 0,5 | 0 - 2,67 | 0 | - | 100 ± 0 | 100 - 100 | 0 ± 0 | 0 - 0 |
|  | Illetes | 10 | 5 | 11,7 | 1,33 ± 0,42 | 0 - 2,67 | 1,07 ± 0,5 | 0 - 2,67 | 60 ± 18,71 | 0 - 100 | 40 ± 18,71 | 0 - 100 |
|  | Migjorn | 10 | 5 | 10,5 | 3,47 ± 1,61 | 0 - 9,33 | 2,4 ± 1,29 | 0 - 6,67 | 60,83 ± 19,17 | 0 - 100 | 39,17 ± 19,17 | 0 - 100 |
|  |  | 20 | 5 | 20,0 | 2,13 ± 0,53 | 1,33 - 4 | 0,53 ± 0,33 | 0 - 1,33 | 85 ± 9,99 | 50 - 100 | 15 ± 10 | 0 - 50 |
|  | Punta de la Gavina | 10 | 5 | 9,3 | 0 | - | 0 | - | - | 0 - 0 | - | 0 - 0 |
|  |  | 20 | 5 | 20,4 | 1,6 ± 0,27 | 1,33 - 2,67 | 0,27 ± 0,27 | 0 - 1,33 | 90 ± 10 | 50 - 100 | 10 ± 10 | 0 - 50 |
|  | Punta Prima | 10 | 5 | 9,9 | 1,07 ± 0,5 | 0 - 2,67 | 0 | - | 100 ± 0 | 100 - 100 | 0 ± 0 | 0 - 0 |
|  |  | 20 | 5 | 18,4 | 0,8 ± 0,53 | 0 - 2,67 | 0 | - | 100 ± 0 | 100 - 100 | 0 ± 0 | 0 - 0 |
| **Ibiza** | Cala Jondal | 10 | 5 | 11,4 | 0,27 ± 0,27 | 0 - 1,33 | 0,27 ± 0,27 | 0 - 1,33 | - | 50 - 50 | - | 50 - 50 |
|  |  | 20 | 5 | 19,4 | 0 | - | 0,67 ± 0,34 | 0 - 1,33 | 0 ± 0 | 0 - 0 | 100 ± 0 | 100 - 100 |
|  | Cala Salada | 10 | 5 | 10,2 | 0 | - | 0,27 ± 0,27 | 0 - 1,33 | - | 0 - 0 | - | 100 - 100 |
|  |  | 20 | 5 | 20,0 | 0,27 ± 0,27 | 0 - 1,33 | 0 | - | - | 100 - 100 | - | 0 - 0 |
|  | Cala Tarida | 10 | 5 | 9,6 | 2,13 ± 1,16 | 0 - 6,67 | 0 | - | 100 ± 0 | 100 - 100 | 0 ± 0 | 0 - 0 |
|  |  | 20 | 5 | 19,9 | 0 | - | 0 | - | - | 0 - 0 | - | 0 - 0 |
|  | Conejera | 10 | 5 | 10,4 | 0,53 ± 0,33 | 0 - 1,33 | 0 | - | 100 ± 0 | 100 - 100 | 0 ± 0 | 0 - 0 |
|  |  | 20 | 5 | 19,7 | 1,87 ± 0,8 | 0 - 4 | 0,27 ± 0,27 | 0 - 1,33 | 75 ± 22,36 | 0 - 100 | 25 ± 22,36 | 0 - 100 |
|  | Moranell | 10 | 5 | 9,8 | 0,27 ± 0,27 | 0 - 1,33 | 0,8 ± 0,53 | 0 - 2,67 | 16,67 ± 10,54 | 0 - 33,33 | 83,33 ± 10,54 | 66,67 - 100 |
|  |  | 20 | 5 | 20,5 | 1,33 ± 0,6 | 0 - 2,67 | 0,27 ± 0,27 | 0 - 1,33 | 88,89 ± 8,61 | 66,67 - 100 | 11,11 ± 8,61 | 0 - 33,33 |
|  | Ses Formigues | 10 | 5 | 9,5 | 0,27 ± 0,27 | 0 - 1,33 | 0,27 ± 0,27 | 0 - 1,33 | 50 ± 31,62 | 0 - 100 | 50 ± 31,62 | 0 - 100 |
|  |  | 20 | 5 | 20,0 | 0,8 ± 0,53 | 0 - 2,67 | 1,33 ± 0,6 | 0 - 2,67 | 37,5 ± 21,41 | 0 - 100 | 62,5 ± 21,41 | 0 - 100 |
|  | Tagomago | 10 | 5 | 10,0 | 0,53 ± 0,53 | 0 - 2,67 | 0,8 ± 0,53 | 0 - 2,67 | 33,33 ± 25,82 | 0 - 100 | 66,67 ± 25,82 | 0 - 100 |
|  |  | 20 | 5 | 18,9 | 0,53 ± 0,33 | 0 - 1,33 | 0,27 ± 0,27 | 0 - 1,33 | 66,67 ± 25,82 | 0 - 100 | 33,33 ± 25,82 | 0 - 100 |
|  | Talamanca | 10 | 5 | 10,9 | 3,47 ± 1,5 | 1,33 - 9,33 | 0 | - | 100 ± 0 | 100 - 100 | 0 ± 0 | 0 - 0 |
|  |  | 20 | 5 | 18,5 | 1,07 ± 0,27 | 0 - 1,33 | 0,27 ± 0,27 | 0 - 1,33 | 80 ± 20 | 0 - 100 | 20 ± 20 | 0 - 100 |
| **Mallorca** | Aucanada | 10 | 5 | 9,5 | 1,6 ± 1,07 | 0 - 5,33 | 0,8 ± 0,33 | 0 - 1,33 | 41,67 ± 22,36 | 0 - 100 | 58,33 ± 22,36 | 0 - 100 |
|  |  | 20 | 5 | 18,8 | 0 | - | 0,27 ± 0,27 | 0 - 1,33 | - | 0 - 0 | - | 100 - 100 |
|  | Cala Fornells | 10 | 5 | 10,2 | 0 | - | 0,53 ± 0,33 | 0 - 1,33 | 0 ± 0 | 0 - 0 | 100 ± 0 | 100 - 100 |
|  |  | 20 | 5 | 19,5 | 0,27 ± 0,27 | 0 - 1,33 | 0,27 ± 0,27 | 0 - 1,33 | 50 ± 31,62 | 0 - 100 | 50 ± 31,62 | 0 - 100 |
|  | Cala Matzocs | 10 | 5 | 10,2 | 3,73 ± 1,22 | 1,33 - 6,67 | 0,27 ± 0,27 | 0 - 1,33 | 96,67 ± 3,33 | 83,33 - 100 | 3,33 ± 3,33 | 0 - 16,67 |
|  |  | 20 | 5 | 20,3 | 3,2 ± 0,68 | 1,33 - 5,33 | 0 | - | 100 ± 0 | 100 - 100 | 0 ± 0 | 0 - 0 |
|  | Cala Ortigues | 10 | 5 | 10,1 | 1,07 ± 0,5 | 0 - 2,67 | 0,8 ± 0,53 | 0 - 2,67 | 62,5 ± 21,41 | 0 - 100 | 37,5 ± 21,41 | 0 - 100 |
|  |  | 20 | 5 | 20,1 | 1,6 ± 1,07 | 0 - 5,33 | 0,27 ± 0,27 | 0 - 1,33 | 90 ± 6,32 | 80 - 100 | 10 ± 6,32 | 0 - 20 |
|  | Cala Tuent N | 10 | 5 | 9,8 | 2,4 ± 1,29 | 0 - 6,67 | 0,27 ± 0,27 | 0 - 1,33 | 91,67 ± 6,45 | 75 - 100 | 8,33 ± 6,45 | 0 - 25 |
|  |  | 20 | 5 | 20,0 | 3,73 ± 0,98 | 1,33 - 6,67 | 0,8 ± 0,33 | 0 - 1,33 | 82,67 ± 7,48 | 66,67 - 100 | 17,33 ± 7,48 | 0 - 33,33 |
|  | Cala Virgili | 10 | 5 | 11,1 | 0,8 ± 0,53 | 0 - 2,67 | 0,27 ± 0,27 | 0 - 1,33 | 66,67 ± 25,82 | 0 - 100 | 33,33 ± 25,82 | 0 - 100 |
|  |  | 20 | 5 | 18,9 | 1,33 ± 0,73 | 0 - 4 | 0,27 ± 0,27 | 0 - 1,33 | 75 ± 22,36 | 0 - 100 | 25 ± 22,36 | 0 - 100 |
|  | Es Caló-Farrutx | 10 | 5 | 11,1 | 5,87 ± 0,68 | 4 - 8 | 1,33 ± 0,6 | 0 - 2,67 | 81,33 ± 8,27 | 60 - 100 | 18,67 ± 8,27 | 0 - 40 |
|  |  | 20 | 5 | 21,7 | 1,07 ± 0,49 | 0 - 2,67 | 0,27 ± 0,27 | 0 - 1,33 | 83,33 ± 12,91 | 50 - 100 | 16,67 ± 12,91 | 0 - 50 |
|  | Foradada | 10 | 5 | 10,4 | 2,13 ± 1,24 | 0 - 6,67 | 0,27 ± 0,27 | 0 - 1,33 | 94,44 ± 4,3 | 83,33 - 100 | 5,55 ± 4,3 | 0 - 16,67 |
|  |  | 20 | 5 | 18,3 | 1,87 ± 0,99 | 0 - 5,33 | 0,8 ± 0,53 | 0 - 2,67 | 66,67 ± 21,08 | 0 - 100 | 33,33 ± 21,08 | 0 - 100 |
|  | Formentor | 10 | 5 | 10,0 | 0,27 ± 0,27 | 0 - 1,33 | 0 | - | - | 100 - 100 | - | 0 - 0 |
|  |  | 20 | 5 | 19,6 | 0 | - | 0 | - | - | 0 - 0 | - | 0 - 0 |
|  | Malgrats | 10 | 5 | 10,7 | 2,4 ± 1,07 | 0 - 5,33 | 0,27 ± 0,27 | 0 - 1,33 | 88,89 ± 8,61 | 66,67 - 100 | 11,11 ± 8,61 | 0 - 33,33 |
|  |  | 20 | 5 | 19,2 | 0 | - | 0 | - | - | 0 - 0 | - | 0 - 0 |
|  | Migjorn integral | 10 | 5 | 9,0 | 8 ± 1,74 | 5,33 - 14,67 | 0 | - | 100 ± 0 | 100 - 100 | 0 ± 0 | 0 - 0 |
|  |  | 20 | 5 | 19,4 | 3,73 ± 0,88 | 1,33 - 6,67 | 0 | - | 100 ± 0 | 100 - 100 | 0 ± 0 | 0 - 0 |
|  | Sa Coma | 10 | 5 | 10,4 | 2,13 ± 0,68 | 0 - 4 | 0,27 ± 0,27 | 0 - 1,33 | 91,67 ± 7,45 | 66,67 - 100 | 8,33 ± 7,45 | 0 - 33,33 |
|  |  | 20 | 5 | 19,3 | 1,07 ± 0,5 | 0 - 2,67 | 0,53 ± 0,33 | 0 - 1,33 | 62,5 ± 21,41 | 0 - 100 | 37,5 ± 21,41 | 0 - 100 |
|  | San Telmo-Dragonera | 10 | 5 | 10,7 | 1,6 ± 0,78 | 0 - 4 | 1,07 ± 0,5 | 0 - 2,67 | 56,25 ± 19,09 | 0 - 100 | 43,75 ± 19,09 | 0 - 100 |
|  |  | 20 | 5 | 18,2 | 2,93 ± 1,07 | 0 - 5,33 | 0,53 ± 0,33 | 0 - 1,33 | 76 ± 19,39 | 0 - 100 | 24 ± 19,39 | 0 - 100 |
|  | Ses Salines | 10 | 5 | 10,0 | 0,27 ± 0,27 | 0 - 1,33 | 0,53 ± 0,33 | 0 - 1,33 | 33,33 ± 25,82 | 0 - 100 | 66,67 ± 25,82 | 0 - 100 |
|  |  | 20 | 5 | 18,1 | 2,67 ± 1,26 | 0 - 6,67 | 0,27 ± 0,27 | 0 - 1,33 | 88,89 ± 8,61 | 66,67 - 100 | 11,11 ± 8,61 | 0 - 33,33 |
| **Menorca** | Aire 1 | 10 | 5 | 9,7 | 3,67 ± 1,33 | 0 - 8,33 | 0,67 ± 0,41 | 0 - 1,67 | 83,33 ± 8,61 | 66,67 - 100 | 16,67 ± 8,61 | 0 - 33,33 |
|  |  | 20 | 5 | 20,2 | 7,33 ± 0,41 | 6,67 - 8,33 | 1 ± 0,41 | 0 - 1,67 | 88,67 ± 4,66 | 80 - 100 | 11,33 ± 4,67 | 0 - 20 |
|  | Alcaufar | 10 | 5 | 10,4 | 7,33 ± 2,56 | 1,67 - 16,67 | 0,33 ± 0,33 | 0 - 1,67 | 98,18 ± 1,82 | 90,91 - 100 | 1,82 ± 1,82 | 0 - 9,09 |
|  |  | 20 | 5 | 20,8 | 2,33 ± 0,67 | 0 - 3,33 | 0,33 ± 0,33 | 0 - 1,67 | 87,5 ± 11,18 | 50 - 100 | 12,5 ± 11,18 | 0 - 50 |
|  | Cala en Porter | 10 | 5 | 10,7 | 1,87 ± 1,24 | 0 - 6,67 | 0 | - | 100 ± 0 | 100 - 100 | 0 ± 0 | 0 - 0 |
|  |  | 20 | 5 | 18,5 | 4 ± 1,19 | 0 - 6,67 | 0,8 ± 0,53 | 0 - 2,67 | 83,33 ± 8,61 | 66,67 - 100 | 16,67 ± 8,61 | 0 - 33,33 |
|  | Cala Rafelet | 20 | 5 | 20,1 | 5,67 ± 0,85 | 3,33 - 8,33 | 0,33 ± 0,33 | 0 - 1,67 | 95 ± 5 | 75 - 100 | 5 ± 5 | 0 - 25 |
|  | Caló des Vi Blanc | 10 | 5 | 10,5 | 1 ± 0,67 | 0 - 3,33 | 0,67 ± 0,41 | 0 - 1,67 | 50 ± 25,82 | 0 - 100 | 50 ± 25,82 | 0 - 100 |
|  |  | 20 | 5 | 21,2 | 2,67 ± 0,41 | 1,67 - 3,33 | 1 ± 0,67 | 0 - 3,33 | 76,67 ± 14,53 | 33,33 - 100 | 23,33 ± 14,53 | 0 - 66,67 |
|  | Fornells | 10 | 5 | 10,8 | 3,73 ± 0,98 | 1,33 - 6,67 | 0,53 ± 0,33 | 0 - 1,33 | 90 ± 6,66 | 66,67 - 100 | 10 ± 6,67 | 0 - 33,33 |
|  |  | 15 | 5 | 14,9 | 0 | - | 0,27 ± 0,27 | 0 - 1,33 | - | 0 - 0 | - | 100 - 100 |
|  | La Mola 1 | 10 | 5 | 11,3 | 0,67 ± 0,41 | 0 - 1,67 | 0 | - | 100 ± 0 | 100 - 100 | 0 ± 0 | 0 - 0 |
|  |  | 20 | 5 | 19,6 | 0 | - | 0,33 ± 0,33 | 0 - 1,67 | - | 0 - 0 | - | 100 - 100 |
|  | La Mola 2 | 10 | 5 | 9,1 | 4,67 ± 1,33 | 0 - 6,67 | 0,67 ± 0,41 | 0 - 1,67 | 86,67 ± 7,3 | 66,67 - 100 | 13,33 ± 7,3 | 0 - 33,33 |
|  |  | 20 | 5 | 19,6 | 7,33 ± 2,39 | 0 - 15 | 2,67 ± 0,85 | 0 - 5 | 63,79 ± 17,34 | 0 - 100 | 36,21 ± 17,34 | 0 - 100 |
|  | Port Maó | 10 | 5 | 7,7 | 3,73 ± 1,48 | 1,33 - 9,33 | 0 | - | 100 ± 0 | 100 - 100 | 0 ± 0 | 0 - 0 |
|  | Sa Farola | 10 | 5 | 9,5 | 3,2 ± 2,55 | 0 - 13,33 | 0 | - | 100 ± 0 | 100 - 100 | 0 ± 0 | 0 - 0 |
|  |  | 20 | 5 | 20,6 | 1,87 ± 0,54 | 1,33 - 4 | 0 | - | 100 ± 0 | 100 - 100 | 0 ± 0 | 0 - 0 |
|  | Sanitja | 10 | 5 | 9,2 | 0,8 ± 0,53 | 0 - 2,67 | 0 | - | 100 ± 0 | 100 - 100 | 0 ± 0 | 0 - 0 |
|  |  | 20 | 4 | 19,0 | 5 ± 0,64 | 4 - 6,67 | 1 ± 0,64 | 0 - 2,67 | 85,42 ± 8,59 | 66,67 - 100 | 14,58 ± 8,59 | 0 - 33,33 |
|  | Sant Esteve | 10 | 10 | 11,3 | 0,93 ± 0,45 | 0 - 4 | 0,27 ± 0,27 | 0 - 2,67 | 87,5 ± 7,91 | 50 - 100 | 12,5 ± 7,91 | 0 - 50 |
|  |  | 20 | 9 | 19,5 | 0,59 ± 0,23 | 0 - 1,33 | 0 | - | 100 ± 0 | 100 - 100 | 0 ± 0 | 0 - 0 |
|  | Son Bou | 10 | 5 | 8,8 | 2,93 ± 1,29 | 0 - 6,67 | 0,27 ± 0,27 | 0 - 1,33 | 95,83 ± 3,73 | 83,33 - 100 | 4,17 ± 3,73 | 0 - 16,67 |
|  |  | 20 | 5 | 19,1 | 2,13 ± 0,68 | 0 - 4 | 1,6 ± 0,98 | 0 - 4 | 66,25 ± 17,64 | 25 - 100 | 33,75 ± 17,64 | 0 - 75 |
|  | Son Saura | 10 | 5 | 9,2 | 6,13 ± 1,55 | 1,33 - 9,33 | 0,8 ± 0,53 | 0 - 2,67 | 92,22 ± 4,84 | 77,78 - 100 | 7,78 ± 4,84 | 0 - 22,22 |
|  |  | 20 | 5 | 21,1 | 3,47 ± 0,54 | 2,67 - 5,33 | 0,53 ± 0,33 | 0 - 1,33 | 89,33 ± 6,86 | 66,67 - 100 | 10,67 ± 6,86 | 0 - 33,33 |
